# Resonant dual-pulse laser ignition technique based on oxygen REMPI pre-ionization

**DOI:** 10.1038/s41598-020-76968-5

**Published:** 2020-11-16

**Authors:** Ciprian Dumitrache, Carter Butte, Azer Yalin

**Affiliations:** 1 Plasma and Radiation Physics, Solid-State Quantum Electronics Lab., National Institute for Laser, 077125 Bucharest, Romania; 2 Department of Mechanical Engineering, Colorado State University, Fort Collins, 80525 USA

**Keywords:** Applied optics, Mechanical engineering, Laser-produced plasmas

## Abstract

This contribution investigates a novel laser ignition method based on a dual-pulse resonant pre-ionization scheme. The first laser pulse efficiently creates initial gas ionization (seed electrons) through a 2 + 1 resonantly-enhanced multiphoton ionization (REMPI) scheme targeting molecular oxygen (λ ~ 287.6 nm). This pulse is followed by a second non-resonant near-infrared pulse (λ = 1064 nm) for energy addition into the gas via inverse bremsstrahlung absorption. The sequence of two pulses creates a laser induced plasma that exhibits high peak electron number density and temperature (n_e_ ~ 8 × 10^17^ cm^-3^ at t = 100 ns and T ~ 8000 K at t = 10 μs, respectively). These plasma parameters are similar to those attained for typical single-pulse near-infrared laser plasmas but with the advantage of substantially lower pulse energy (by factor of ~ 2.5) in the dual-pulse REMPI case. A combustion study focusing on ignition of propane/air mixtures shows that the dual-pulse REMPI method leads to an extension of the lean flammability limit, and an increase in combustion efficiency near the lean limit, as compared to laser ignition with a single NIR pulse. The measurement results and observed gas dynamics are discussed in the context of their impact on combustion applications.

## Introduction

The ever-increasing push for energetically efficient and environmentally friendly energy conversion systems, in particular combustion technologies, has driven the interest in laser ignition (LI) as an alternative to conventional ignition systems. LI systems are potentially attractive in this regard, for example, when applied to industrial reciprocating natural gas engines, LI has shown reduction in NO_x_ emissions and extension of the lean limit^1–3^. The technology is of interest to a variety of combustion applications including reciprocating engines, aero-turbines, and rocket engines^4–10^. System level features of LI, as contrasted to conventional igniter technology, include: the lack of electrodes (which tend to act as heat sinks) within the combustion chamber, the ability to achieve fine control over ignition timing, flexibility in locating the ignition source (based on focusing optics), and potentially systems with favorable cost, packaging and reliability parameters.

Recent approaches towards LI have examined dual (or multiple) pulse laser plasmas based on pre-ionization, i.e. where a first laser pulse primarily ionizes the gas (to produce seed electrons) and then subsequent pulse(s) primarily heat and add energy to the plasma gas kernel via inverse Bremsstrahlung (IB)^11,12^. In general, short wavelength is better suited for the preionization (owing to more efficient multi-photon ionization) while longer wavelength radiation is better suited for energy addition (owing to more efficient IB). The preionization approach relies on synergistic effects between the two pulses, in particular the increased absorption of the second pulse caused by the preionization. This is distinct from multi-pulse approaches (usually at a single-wavelength) where the individual pulses each independently cause breakdown^13,14^ Experimentally, Yalin et al. showed that preionization due to an ultraviolet (UV) 266 nm beam in air reduces the breakdown requirement of an overlapped near-infrared (NIR) energy addition beam at 1064 nm^12^. When applied to combustion, Dumitrache et al. showed that the dual-pulse preionization scheme allowed ignition of leaner propane-air mixtures as compared to single-pulse ignition with the same pulse energy^15^. Subsequent modeling work has further elucidated aspects of the pre-ionization laser plasmas. A computational fluid dynamics (CFD) analysis has shown the role of plasma-driven gas dynamics to enhance the early kernel growth, in particular an ability to tailor the resulting laser plasma (by adjusting the axial offset of the two laser pulses) to either suppress or admit the typical 3rd lobe feature which in turn is linked to the ability to ignite lean mixtures^16^. Kinetic modeling has examined nonequilibrium and electron dynamics in the dual-pulse preionization laser plasmas^17^.

Specifying required plasma parameters for ignition in practical applications is challenging owing to the different possible fuel mixtures and flowfields (e.g. ignition of reciprocating engines versus aero-turbines versus scramjets). The ignition kernel volume, in particular, is expected to be of great importance in combustion devices (e.g. some fuel-injected engines) that exhibit poor mixing of the fuel-and-air^18^ since the larger volume is statistically more likely to overlap ignitable (well-mixed) regions. Our past work has shown larger kernel volumes (more elongated along the optical axis) when preionization is employed^19^. In general, high temperature will benefit ignition owing to promoting chain initiation reactions (rates exponential with temperature)^20^, and higher electron density and temperature can increase the production of radicals and metastables from electron impact reactions^21^. However, the situation is also complicated by the associated gas dynamics, in particular fluid entrainment and vortex formation/ejection, which has also been shown to play an important role in early kernel growth^22^. An example of translating to applications is provided by past studies in a rapid compression machine where we showed successful ignition of methane–air mixtures over a range of equivalence ratios with NIR plasmas^3^. While the present work makes the first examination of the dual-pulse REMPI approach in quiescent, room temperature mixtures, future work should include detailed parametric studies tailored to specific applications.

In the present contribution, we investigate the use of a resonant preionization step, specifically the use of oxygen resonant enhanced multiphoton ionization (REMPI) at ~ 287 nm, in place of the 266 nm excitation. As will be shown herein, the resonant scheme allows more efficient electron generation (with the preionization) for much lower total pulse energy (e.g., we obtain n_e_ = 1.3–3.7 × 10^17^ cm^−3^ using a 3 mJ REMPI pulse compared to n_e_ = 2.5–6.1 × 10^16^ cm^−3^ using a non-resonant (1064 nm) 20 mJ pulse with the same beam waists). When the REMPI and NIR pulses are combined, the resonant dual-pulse scheme provides similar gas temperatures and ignition characteristics as compared to the 266/1064 nm scheme but with overall lower energy requirements (E_REMPI+NIR_ = 40 mJ vs. E_UV+NIR_ = 60 mJ). In general, the use of UV wavelengths (harmonic generation) is less energetically efficient on the laser source side. This aspect (along with size, cost, chance of optical damage) should be evaluated at the system level while keeping in mind that in many combustion applications the power of the ignition source is negligible compared with the thermal power of the flame^23^.

**Figure 1:**
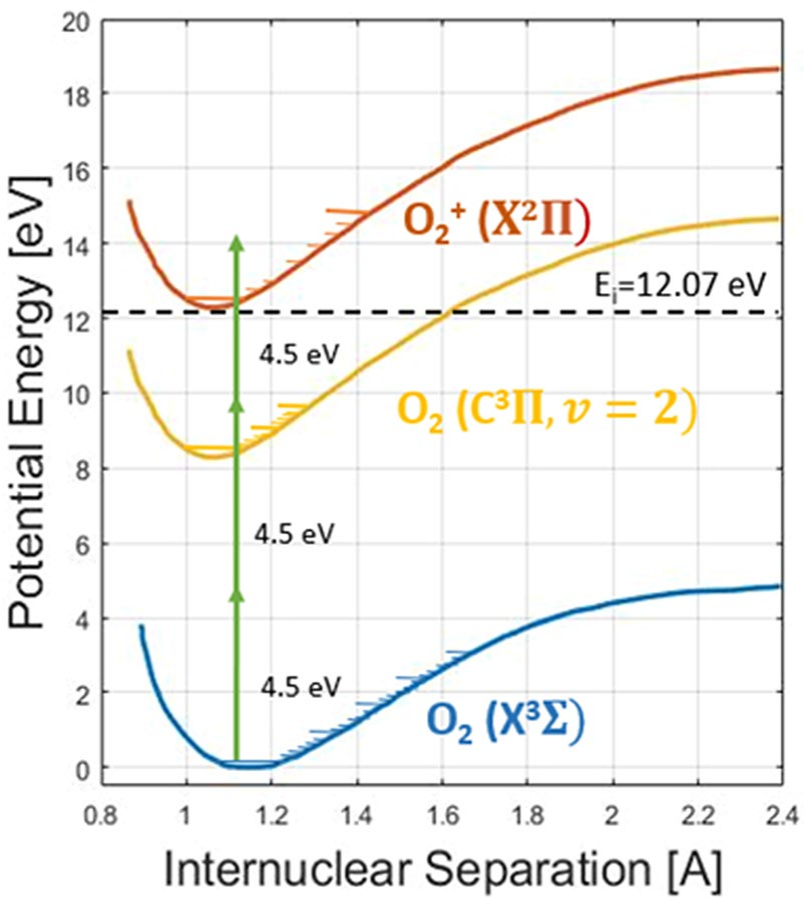
Simplified energy level diagram of molecular oxygen showing the targeted 2 + 1 REMPI scheme (green arrows).

Figure 1 shows a simplified energy level diagram for molecular oxygen including the 2 + 1 REMPI transition of interest. In the 2 + 1 notation, the 2 corresponds to the 2-photon excitation step from the oxygen ground state (X^3^Σ^−^_g_ (ν″ = 0,J″)) to the intermediate state (C^3^Π_g_(ν′ = 2,J′)) which, when excited by a single additional photon (+ 1) is finally excited to an ionized level of molecular oxygen (O_2_^+^ X^2^Π). At high laser intensities, any molecule reaching the intermediate state will also be subsequently ionized such that, as investigated herein, the overall ionization rate scales as the square of the laser intensity (i.e. step-wise ionization is limited by the 2-photon step)^24^. In terms of laser plasma formation, this REMPI transition has been investigated by Adams et al. for breakdown and guiding of discharges^25^ and for thermometry based on microwave scattering from the laser plasma^26,27^ . At high laser intensities effects of anomalous laser absorption can also become important^28,29^.

The remainder of the paper focuses on our investigation of laser plasma formation and ignition using REMPI pre-ionization. We first examine the electron generation (preionization) as a function of laser wavelength in the vicinity of the REMPI transition and confirm resonant 2 + 1 excitation. Next, through the use of Rayleigh and Thomson scattering diagnostics, we consider the temporal profiles of gas temperature and electron density for the dual-pulse preionization plasmas (287 nm followed by 1064 nm pulses). We find that peak temperature and electron density is quite comparable to the corresponding values for single-pulse excitation, but with considerably lower (total) pulse energy in the dual-pulse REMPI case. In terms of ignition, the dual-pulse REMPI case extends the lean limit for propane-air ignition when compared to a single laser pulse (and again with lower pulse energy). The final investigation examines the gas dynamics and vorticity generation in the laser induced plasma revealing the important role of the 3rd lobe in the flame growth. Conclusions and outlook for future research are also presented.

## Results and discussion

### Preionization of air by resonant enhanced multiphoton ionization of oxygen

**Figure 2:**
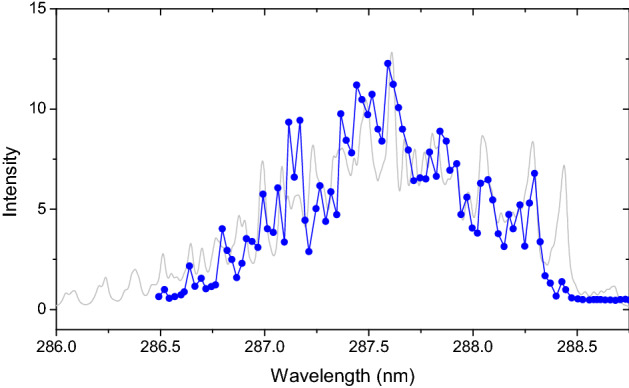
Thomson scattering signal from oxygen (T = 300 K, P = 1.0 atm) as function of pre-ionization laser wavelength. Blue symbols and lines are from experiment while the grey line is simulation (see text).

The first set of experiments was to confirm the role of the oxygen REMPI in the preionization step based on our experimental scheme using focused nanosecond lasers in air (see “Methods” section for full setup). To this end, we have studied both the wavelength- and energy- dependence of the laser preionization step. We employ laser Thomson scattering (with an additional probe laser—see “Methods” section) as a diagnostic for plasma density^30^. Briefly, laser Thomson scattering yields a scattering spectrum due to elastic scattering of photons by electrons which, in the incoherent (non-collective) regime (α << 1), yields a Gaussian spectrum with amplitude proportional to electron density (and probe laser power) and width proportional to the electron temperature. However, for all the scattering measurements reported here a wavelength integrated scattering signal was used. Figure 2 shows the resulting Thomson scattering signal (indicative of electron generation) as a function of the wavelength of the preionization laser. For these data, the Thomson scattering signals were recorded immediately after the end of pre-ionization pulse (~ 1 ns after the pulse, when the signal strength was maximum). Also shown in Fig. 2 is a simulated REMPI spectrum for the same transition based on the model described by Wu et al.^27^ Note that the spectrum here refers to the dependence of the full (wavelength-integrated) Thomson signal as a function of the wavelength of the REMPI laser (not the spectrum of Thomson scattered light). The modeled spectrum requires detailed knowledge of the two-photon line strengths and includes Voigt convolution of laser linewidth with the thermal and natural line-broadening. The spectrum is due to the rotational structure of the vibronic transition from the molecular oxygen ground state to the intermediate state, i.e. C^3^Π_g_(ν′ = 2,J′) ←← X^3^Σ^−^_g_ (ν″ = 0,J″), as this is the rate-limiting step of the 2 + 1 step-wise ionization. The reasonable agreement between model and experiment, including the fact that we observe no Thomson signal when detuned from the transition, confirm that ionization is via REMPI, as opposed to via (non-resonant) multi-photon ionization (MPI) and/or EAI. The discrepancies that are present are likely due to a combination of: (1) laser pulse energy (and beam quality) variations over the broad scanning interval and (2) the need to further tune the detailed line strengths and linewidths in the model. The REMPI assumption was further examined by measuring the dependence of the Thomson scatter signal on the energy of the pre-ionization pulse (at fixed wavelength of ~ 287.62 nm). These experiments showed that the Thomson scatter intensity varied with the square of the intensity of the pre-ionization beam (i.e. *n*_e_ ~ *I*^2^) which further confirms plasma excitation via the 2 + 1 REMPI^31^. This result can be contrasted with similar studies (based on optical emission as a proxy for ionization) where, for 266 nm ionization, we found a cubic dependence (i.e. *n*_e_ ~ *I*^3^) indicative of oxygen MPI (based on *E*_*I,O2*_ = 13.6 eV and *E*_*photon,266 nm*_ = 4.66 eV)^19^. In summary, these tests confirm the role of the oxygen REMPI pre-ionization in the first step of our dual-pulse scheme. For all subsequent experiments, the wavelength of the REMPI beam was set to ~ 287.62 nm at the approximate peak of the spectrum.

### Electron density of dual-pulse REMPI plasmas

**Figure 3:**
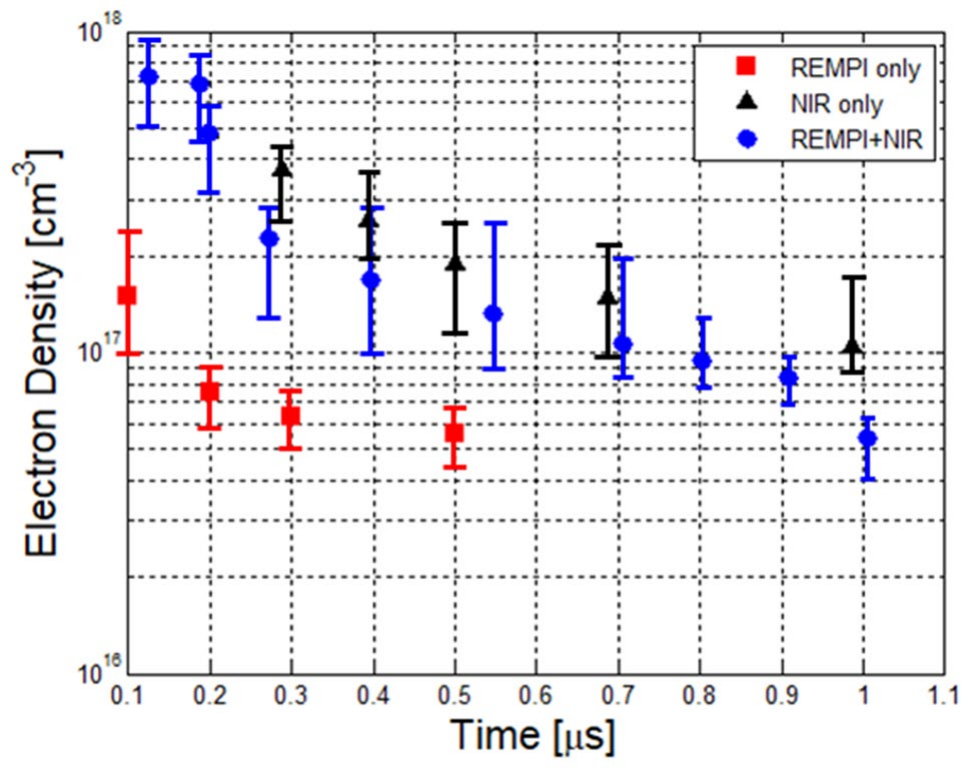
Temporal profiles of electron number density after breakdown for three different laser-generated plasmas: O_2_ REMPI: E_287 nm_ = 3 mJ (red), single-pulse NIR: E_1064 nm_ = 95 mJ (black) and dual-pulse REMPI: E_287+1064 nm_ = 40 mJ (blue).

1-D Rayleigh and Thomson scattering measurements were performed to determine the plasma temperature and electron number density profiles. Figure 3 shows the resulting electron density during the post-discharge phase (0.1–1 μs after the pulses) for three cases: O_2_ REMPI pulse on its own (*λ* = 287.6 nm, E = 3 mJ), single NIR pulse (*λ* = 1064 nm, E = 95 mJ), and the dual-pulse REMPI (*λ* = 287.62 nm, E = 3 mJ and *λ* = 1064 nm, E = 37 mJ). The measurements were conducted in a pressure cell filled with zero-grade air at p_0_ = 1 bar and T_0_ = 298 K. Even with the relatively low REMPI pulse energy a significant degree of ionization is still achieved. For example, we find electron number density of n_e_ = (1.9 ± 0.8) × 10^17^ cm^−3^ at 100 ns after the pulse). In contrast, the non-resonant near-infrared (NIR) pulse does not achieve measurable ionization (breakdown) at such low energies. Indeed, for single-pulse NIR, a pulse energy of 95 mJ was adopted for testing as it was the lowest energy for which consistent (visible) breakdown was achieved. However, once formed, the NIR plasma is found to be almost an order of magnitude denser as compared to the (lower pulse energy) REMPI case, for example n_e_ = (8.3 ± 1.3) × 10^17^ cm^-3^ at 110 ns after the pulse. These results are largely explained by the different mechanism involved in non-resonant breakdown at 1064 nm where most of the free-electrons are generated by electron avalanche ionization (as opposed to MPI or REMPI) which also results in a stronger threshold behavior^19^. For the dual-pulse REMPI case, the resulting plasma is similarly dense as compared to the single-pulse NIR pulse but using approximately 2.5 times less total pulse energy, i.e. 40 mJ (= 3 mJ + 37 mJ) total for dual-pulse REMPI versus 95 mJ for single-pulse NIR. The fast decay in electron density is explained by two main factors: kinetics (rapid recombination and attachment) and gas-dynamics (both experiments and simulations showed that the hot kernel expands very quickly during the first microsecond after plasma formation thus leading to a decrease in density^22^). In all cases presented in Fig. 3, the vertical error bars are due to the uncertainty in discriminating between the Thomson and Rayleigh scattering contributions at early times.

### Temperature of single- and dual-pulse REMPI plasma

**Figure 4:**
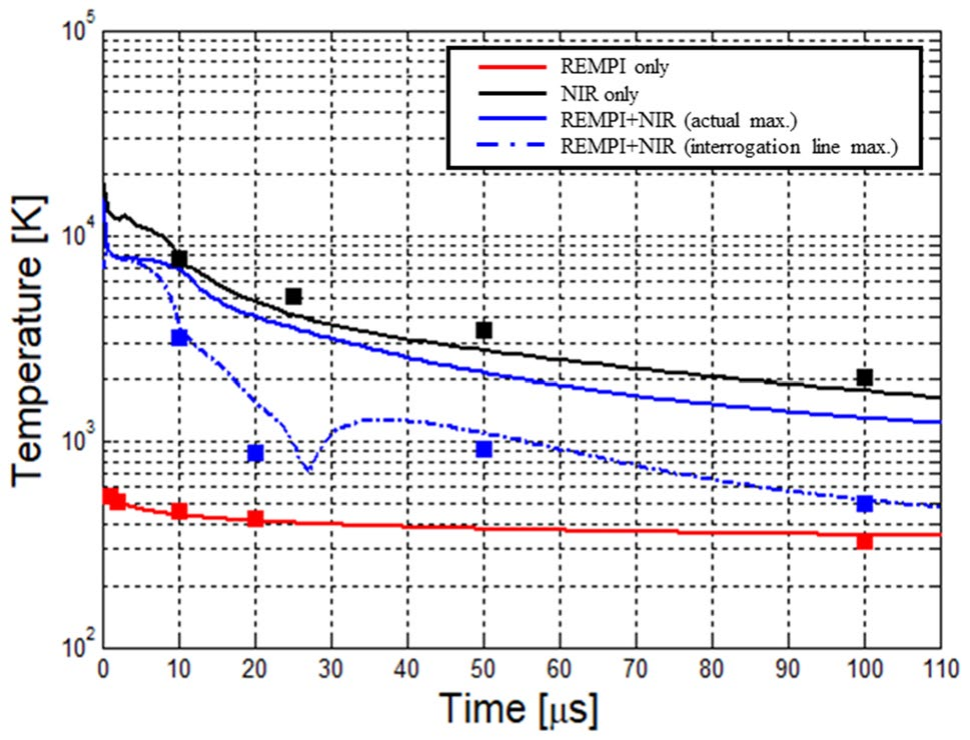
Gas temperature as a function of time after breakdown for three different types of laser-generated plasma: 2 + 1 O_2_ REMPI (red), NIR (black) and combined REMPI + NIR (blue). Gas temperature profiles predicted by CFD modeling are shown with lines.

Gas temperature measurements are performed from Rayleigh scattering signals using the same experimental setup as was used for electron density (including the same pulse energies). The Rayleigh signals can only be accurately analyzed (with no Thomson interference) at sufficiently late times after the plasma forming laser pulses^11,19,32^. Figure 4 presents gas temperature measurements begininng 10 μs after the excitation. For the earlier delays, temperature is inferred using numerical simulations of the hot plasma kernel (see “Methods” section for details). The CFD-predicted temperature profiles are shown with solid lines in Fig. 4. The tempeature profile shows that the 2 + 1 O_2_ REMPI plasma is extremly cold (given the degree of ionization) with a maximum measured temperature of ~ 530 K at 1 μs after the discharge. This is to be expected in a rezonant ionization process where most of the laser energy goes directly into ionization, in this case forming $O_{2}^{ + } \left( X \right)$ and free electrons, as opposed to joule heating induced by IB. For the NIR breakdown case, we find considerably higher temperatures in excess of 8300 K at 10 μs after the pulse (~ 15,000 K predicted by CFD at t = 0, i.e. at the end of the excitation pulse). As with the electron density results reported above, the dual-pulse REMPI allows similar plasma parameters (temperature in this case) when compared to single-pulse (NIR) but with only ~ 40% of the total pulse energy. The temperatures plotted in Fig. 4 correspond to the maximum temperature of the laser plasma at the given time. For the single-pulse experiments (REMPI only and NIR only) this was achieved by iteratively moving the Rayleigh probe beam at each time delay; however, owing to the potentially more complex flowfield, the dual REMPI + NIR case used a fixed measurement location. For this reason, we present two curves for the REMPI dual-pulse case in Fig. 4: the maximum temperature at a fixed location across the kernel shown in dashed blue line (data which matches well with the experimental data) and the overall peak plasma temperature from CFD. The sudden increase in temperature between 30 and 40 μs (dashed blue line) is due to strong gas dynamics associated with the motion of a hot lobe towards the side of the incoming laser during gas cooling phase. This peak does not appear in the other three curves which track overall maximum temperature because the frontal lobe never becomes the hottest part of the kernel. Finally, each experimental point includes a vertical error bar of ± 20 K, mostly due to shot-noise, but the error bars do not show on the semi-logarithmic plot.

### Ignition testing and combustion efficiency

**Figure 5:**
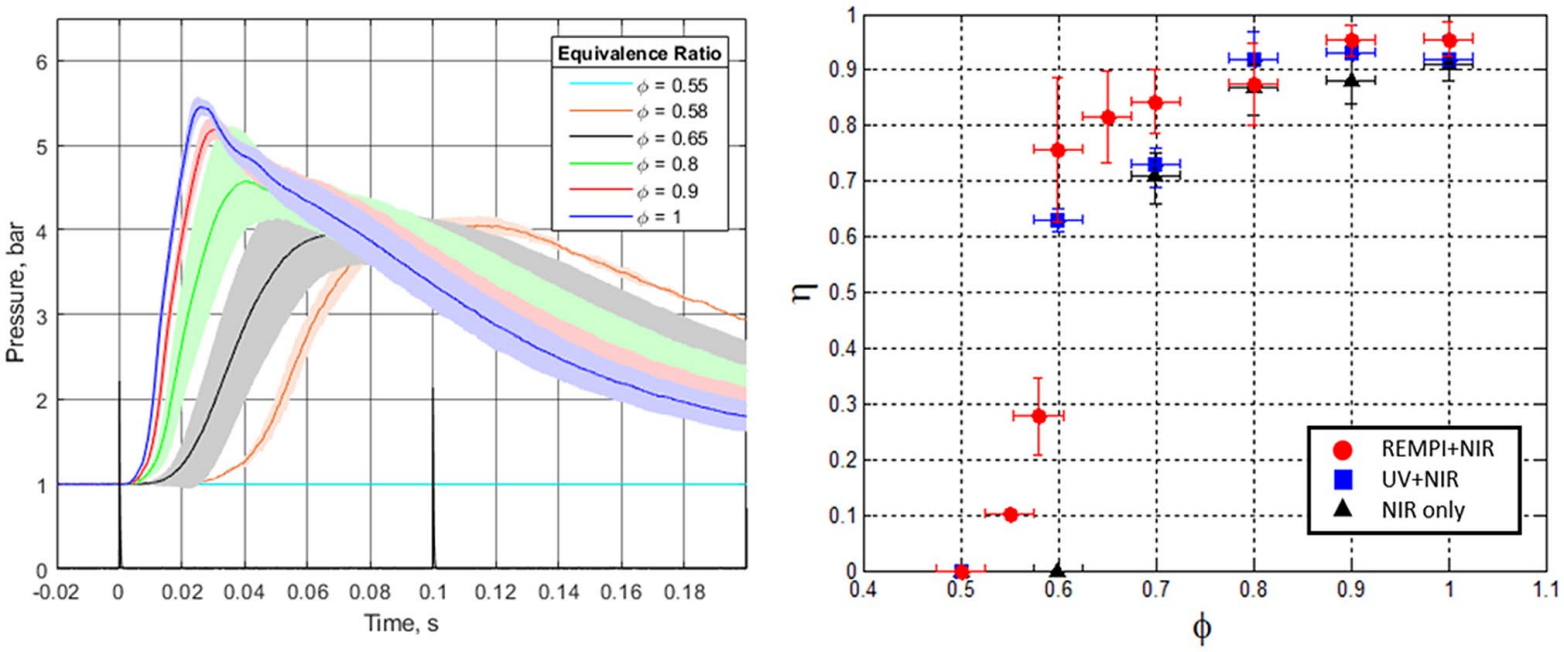
Left: Pressure traces due to dual-pulse REMPI ignition of propane–air mixtures. The different curves correspond to different equivalence ratios. Right: Combustion efficiency for 3 cases of laser ignition of propane–air mixtures: dual-pulse REMPI (red), non-resonant dual-pulse (blue), and single-pulse NIR (black). Each point represents the average of at least three tests and the error bars are ± 1 standard deviation.

Laser ignition experiments were conducted in a pressure cell with mixtures of propane-air at initial conditions of T_0_ = 323 K and p_0_ = 1 bar. Combustion events were monitored based on the temporal evolution of pressure inside the cell. Pressure traces corresponding to ignition of C_3_H_8_/Air mixtures via the combined REMPI + NIR pulses are shown in Fig. 5-left below. The end-time of the plasma forming laser pulses defines t = 0 in the pressure traces. After a certain delay (which depends on the mixture’s equivalence ratio, ϕ), a pressure rise is observed in the mixtures that ignited successfully. The pressure rise is due to heat release by chemical reactions which raise the temperature of the fixed-volume cell. As leaner mixtures are tested, the peak pressure reduces. This is mainly a consequence of the reduction in mixture reactivity. However, for the ultra-lean cases (ϕ < 0.7), a decrease in combustion efficiency is also observed. This is shown in Fig. 5-right, where the combustion efficiency, i.e. the fraction of starting fuel energy that gets converted into heat, η, is plotted for each equivalence ratio.

We contrast the performance of the dual-pulse REMPI (*λ* = 287.62 nm, E = 3 mJ and *λ* = 1064 nm, E = 37 mJ) ignition scheme with past (baseline) measurements of combustion efficiencies for single-pulse NIR (*λ* = 1064 nm, E = 95 mJ) and non-resonant dual-pulse (*λ* = 266 nm, E = 20 mJ and *λ* = 1064 nm, E = 40 mJ)^15^ as also shown in Fig. 5-right. The experimental results indicate the dual-pulse REMPI technique can ignite leaner mixtures as compared to single-pulse NIR (ϕ_Min_ = 0.55 for the dual-pulse REMPI and ϕ_Min_ = 0.7 for single-pulse NIR, respectively), as well as achieving higher combustion efficiency for the leanest ignited mixtures. Additionally, the REMPI + NIR technique exhibits higher combustion efficiency towards the lean limit as compared to the non-resonant dual-pulse. Ignition using the REMPI pulse on its own (with no accompanying 1064 nm beam) was unsuccessful due to the low temperature generated by the resonant plasma (Fig. 4). The shaded bands around the pressure traces in Fig. 5-left correspond to ± 1 standard deviation after averaging multiple ignition events. In general, the pressure traces become more variable as the equivalence ratio is reduced. Finally, the vertical error bars in the combustion efficiency plot correspond to one standard deviation in calculated combustion efficiency based on each individual pressure trace. The horizontal error bars for equivalence ratio correspond to the uncertainty in the pressure gauge reading used for mixing the gases.

### Gas dynamics of dual-pulse REMPI plasmas

**Figure 6:**
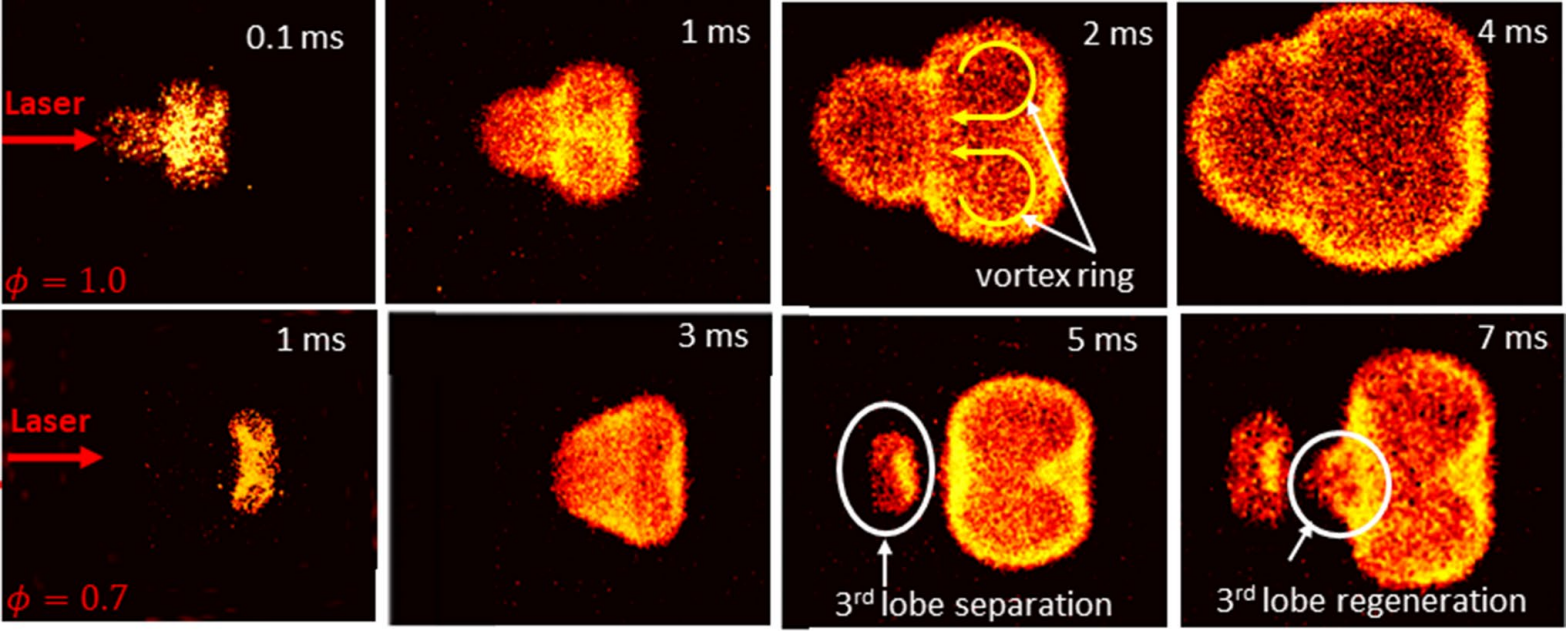
OH* chemiluminescence images showing the early flame kernel dynamics for stoichiometric: ϕ = 1.0 (top) and lean: ϕ = 0.7 (bottom) mixtures due to laser ignition with the dual-pulse REMPI configuration.

The flame dynamics induced by the laser plasma kernels are investigated based on OH* chemiluminescence images. The image sequences shown in Fig. 6 are due to ignition of C_3_H_8_/Air mixtures at stoichiometric ratio of ϕ = 1.0 (top) and a fuel-lean condition of ϕ = 0.7 (bottom) using the dual-pulse REMPI laser technique (*λ*  = 287.62 nm, E = 3 mJ and *λ* = 1064 nm, E = 37 mJ). At stoichiometric conditions, the flame dynamics exhibit similar behavior as has been observed in the post-discharge of NIR laser plasmas^33–38^. Specifically, one observes an asymmetric toroidal flame structure having two side lobes (due to slicing of the actual toroidal feature with the 2-D section) and a third (frontal) lobe that tends to propagate towards the laser source.

Interestingly, lean mixtures appear to exhibit a rapid quenching of the third lobe within the first few hundred microseconds (note that already at 1 ms in Fig. 6 below, the frontal lobe is missing from the images). Nonetheless, vorticity generated inside the toroid continues to entrain combustible fluid through its hot center and back towards the laser source. This effect appears to cause multiple lobe re-ignition and separation events during the combustion event. The detachment and extinction of the 3^rd^ lobe has been previously observed in single-pulse (NIR) laser breakdown^15,39^. However, the regeneration of the third lobe at later times has not, to our knowledge, been reported before. This might suggest that the dual-pulse REMPI breakdown generates stronger (more persistent) vorticity resulting in the observed gas-dynamics in the lean mixtures case, as compared to other LI modes.

## Summary

The current study presents a new laser ignition technique based on resonant pre-ionization of oxygen molecules at ~ 287.6 nm. For subsequent energy addition, we employ a dual-pulse sequence where the first 287.6 nm resonant pulse is followed by a NIR 1064 nm pulse. The presence of resonant photoionization was confirmed via rotationally resolved spectra for the two-photon excitation of the $C^{3}\Pi _{g} \left( {v^{\prime} = 2} \right) \leftarrow X^{3}\Sigma _{g}^{ - } \left( {v^{\prime\prime} = 0} \right)$ transition using laser Thomson scattering. The plasma was further characterized using Thomson and Rayleigh scattering to determine the electron number density and gas temperature evolution after the spark. The REMPI plasma was compared with the plasma due to a typical NIR single-pulse (1064 nm) and it was noted that a significant degree of ionization can be obtained using only a fraction of the laser energy. Gas heating induced by the REMPI plasma was noted to be minimal; however, when combined with a lower energy NIR pulse, one could get similar temperatures to a NIR breakdown plasma by only using ~ 40% of the total laser energy. Finally, ignition testing with the dual-pulse REMPI technique showed the possibility of increased combustion efficiency at lean conditions as compared to other laser ignition schemes. Ignition testing also showed that the lean limit can be extended from ϕ = 0.7 (for single-pulse NIR breakdown) to ϕ = 0.55 using the dual-pulse REMPI technique.

## Methods

### Optical layout

**Figure 7:**
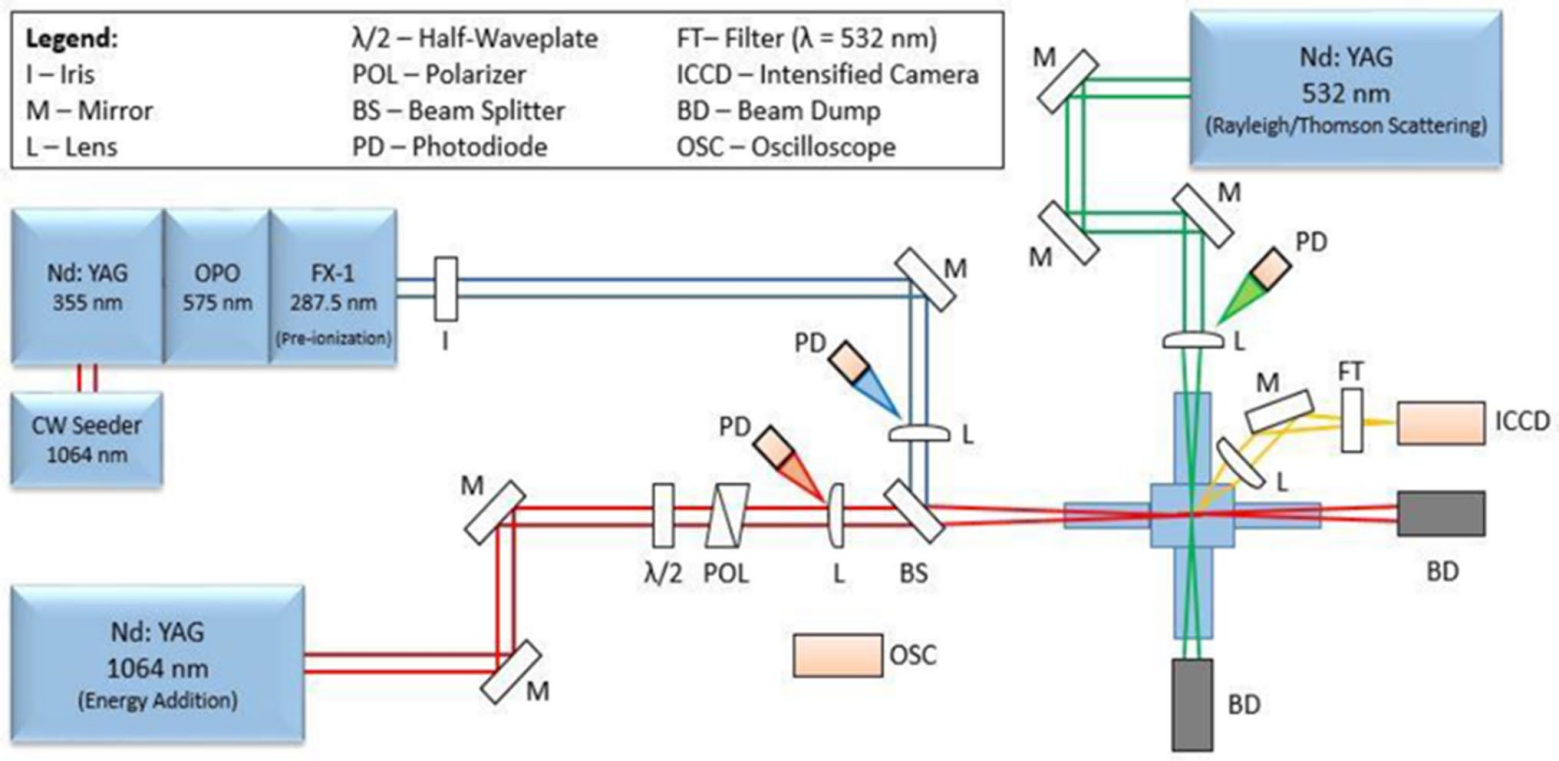
Optical layout used for the laser ignition experiments. Dual-pulse REMPI experiments uses overlapped beams from the 287 nm and 1064 nm laser outputs. An additional 532 nm beam is for scattering diagnostics.

The experiments were conducted using the basic setup shown in Fig. 7. The REMPI pre-ionization beam is due to the (*λ* ~ 287 nm) output (doubled-signal beam) from an optical parametric oscillator (OPO) laser system (Continuum Sunlite). The OPO is pumped by a seeded Nd:YAG laser (Continuum Powerlite 8010). For dual-pulse schemes, the energy addition pulse was the fundamental output (*λ *= 1064 nm) from a second Nd:YAG (New Wave Gemini PIV). The energy of this laser could be changed by a variable attenuator comprised of a half-wave plate and polarizer pair. The two beams are spatially overlapped (with precision ~ 10 ${\mu m}$) using a beam splitter and focused inside the combustion chamber using two *f* = 300 mm plano-convex lenses (one in each beam path). To vary the offset between the two beams in the axial direction (i.e. along the beam propagation direction), a translation stage was used to move the focusing lens of the 1064 nm beam. Fine-tuning of the overlap of the two beams (REMPI and NIR) is achieved by minimizing the beam energies required for plasma formation in lab air. For the non-resonant UV + NIR dual-pulse measurements, the UV pulse was obtained from the same Continuum Powerlite 8010 by replacing the third harmonic crystal with a fourth harmonic crystal (266 nm) and overriding the OPO path. For the Rayleigh/Thomson scattering experiments a third probe beam, due to the 532 nm output of another Nd:YAG laser (Spectra-Physics Quanta-Ray), was brought in orthogonally to the two plasma-formation (287 nm and 1064 nm) lasers using a set of dielectric mirrors and a plano-convex lens (f = 250 mm). A series of photodiodes and energy meters were used to monitor laser pulse durations and pulse energies of each beam leg (i.e. 287 (or 266) nm and 1064 nm) both before and after passage through the focal region and combustion chamber. The axial overlap and the beam diameters at the focus were measured using a beam profiler (Ophir SP503). The beam characteristics for each laser beam used are summarized in Table 1. Beam intensities can be determined from pulse energy, duration, and beam diameter. For a full description of the laser beam parameters, the M^2^ beam quality factor is also provided in Table 1. Spatial- and temporal- intensity fluctuations within multi-mode beams can influence the efficiency of the multiphoton ionization process^40^.

**Table 1:** Laser parameters used in the experiments.

Laser type	Wavelength (nm)	Pulse duration (ns)	Waist diameter (μm)	M^2^ factor	Energy (mJ)
REMPI	286–290	7	150	3.4	3
UV	266	7	150	1.9	20
NIR	1064	10	150	3.2	95
Thomson/Rayleigh scattering	532	12	170	2.1	5

### Thomson/Rayleigh scattering measurements

**Figure 8:**
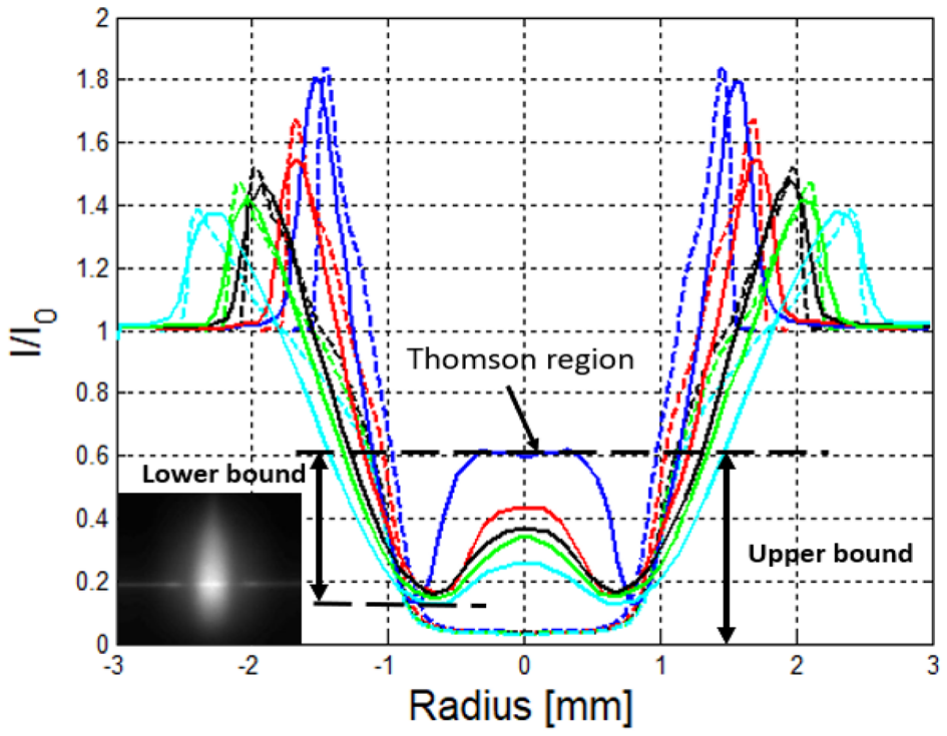
1-D profiles of combined Rayleigh/Thomson scatter signal along the direction perpendicular to the plasma formation laser beams. The scattering intensity has been normalized to a reference condition of Rayleigh scattering in air. The signal rise near the center at early times (small delays) is attributed to Thomson scattering from free electrons. The insert shows a raw ICCD image from the plasma (before background subtraction and normalization) at delay of 100 ns. Experimental profiles are shown with solid lines and CFD-generated best-fit profiles are shown with dashed lines.

Diagnostic measurements of gas temperature and electron density of the laser plasmas were performed by Rayleigh and Thomson scattering. Details on these methods have been presented in our past publications^19,41,42^ and here we summarize the essential features. The probe beam is nominally aligned to pass through the center of the laser plasma to allow 1-D (line) measurement of (combined) Rayleigh and Thomson scattering from the laser plasma (see Fig. 8). Baffles are introduced into the chamber to reduce stray light scattering from the chamber windows. The plasma is imaged using a broadband collection lens (f = 75 mm) and a silver-coated steering mirror into an ICCD camera (pco DICAM pro) which observes the scattering volume orthogonally from above. A bandpass filter at 532 nm (Thorlabs FL532-10) is used to suppress background light. For scattering measurements, 768 images are averaged at each point of interest (3 sets of 256 images averaged on the camera chip) using 2 × 2 binning, resulting in a detector array of 513 × 460 pixels.

Each scattering measurement is based on a ratio-normalization approach whereby scattering at a given measurement condition (i.e., at some known delay relative to laser plasma formation) is divided by the scattering from a reference condition (i.e,. scattering from air at ambient temperature and pressure). The following relations are used to obtain the plasma electron number density and temperature at each pixel in the images:

1 $$n_{e} = n_{0} \frac{{I_{0} - I_{B} - I_{P} + I_{D} }}{{I - I_{B} }}\frac{{\sigma_{R} }}{{\sigma_{T} }}$$

2 $$T = T_{0} \frac{{I_{0} - I_{B} }}{{I - I_{B} }}\frac{{\sigma_{R} }}{{\sigma_{R0} }}\left( T \right)$$

where n_e_ is the electron number density, n_0_ is the gas density corresponding to 1 bar, T represents the plasma temperature, T_0_ is the reference ambient temperature, I is the Rayleigh signal with plasma turned on, I_0_ is the intensity at reference conditions (no plasma), and I_B_, I_P_, and I_D_ correspond to background counts, plasma luminosity counts, and dark counts, respectively. This approach automatically normalizes for variations in laser spatial intensity along the beam. Finally, the maximum electron number density (or temperature) is extracted from the images by averaging a 4 × 4 pixel matrix around the maximum along the direction of the probe beam.

Depending on the delay time, one can infer the gas temperature via Rayleigh scattering or electron density via Thomson scattering. Both measurements are based on previously published data for the Thomson, $\sigma_{T}$, and Rayleigh, $\sigma_{R}$, scattering cross-sections^43^ where the ratio approach serves to cancel out constants such as the light collection solid-angle and detector efficiency. The Thomson measurement uses an upper- and lower-bound to yield the corresponding bounds of the electron density estimate. These bounds are shown in Fig. 8 and also described in detail in our previous work^19^. Shot noise (caused by photon counting) is another contribution to the uncertainty. This type of error is more prevalent at the early times when plasma luminosity has to be subtracted from the images (see Eq. 1) but it is still below 10% of the total uncertainty. For Rayleigh scattering measurements shot noise is the major contributor to the error bars shown in Fig. 4. Another potential source of uncertainty is the change in Rayleigh cross-section. In this work, the plasma’s Rayleigh cross section dependence on temperature is taken into account under the assumption of thermal plasma kernel as explained in some of our previous works^42–44^. In this case, the plasma species concentration is determined using a chemical equilibrium code for a given temperature and the mixture’s Rayleigh scattering is thus determined. We have shown previously that, even if the temperature correction to the Rayleigh scattering cross-section is completely neglected, the error on temperature is less than 5% at temperature below 8500 K^44^. Moreover, the error decreases as the plasma cools down.

In some cases, we also employ a computational fluid dynamics (CFD) model to analyze the temperature evolution of the laser plasmas. The CFD simulations are performed using a Riemann solver developed in-house based on the Navier–Stokes equations^16,45,46^. Previous work by the authors has demonstrated that the initial (end-of-the pulse) state of the NIR breakdown kernel can be obtained iteratively by fitting the experimental 1-D scattering profiles at early times as shown in Fig. 8. We remark that this CFD analysis is useful in determining the gas temperature for t < 10 μs, when uncertainties in the Rayleigh cross-sections as well as the concomitant presence of both Thomson and Rayleigh scattering renders the experimental measurement of temperature very challenging. To select the best initial (end-of-the pulse) conditions, we require that both the location and the amplitude of the blast wave peak should agree, between simulated and measured Rayleigh profiles, to better than 5%. An example of the best fit is shown in Fig. 8 below with dashed lines for the breakdown of the NIR pulse. The corresponding NIR plasma kernel (at its very earliest formation time) is an ellipsoid with major axis of 1.5 mm, and minor axis (in the radial direction) of 0.3 mm, yielding an overall volume of 0.5 mm^3^. By comparison a dual-pulse kernel takes an almost cylindrical shape with a height of 2 cm and a radius of 0.3 mm, yielding an overall volume of 5.5 mm^3^. More details about the CFD model and the fitting procedure can be found in our previous work^22^.

In particular, CFD is introduced in Fig. 4 to correct the experimental temperature measurements for the REMPI dual pulse case. Due to the strong gas dynamics effects involved in the kernel’s post-discharge development, the scattering probe beam has to be re-adjusted at each time delay in order to find the location of maximum temperature. Instead, here the beam was kept at a fixed location that corresponded to the highest Thomson signal at the early time delays (those reported in Fig. 3) and temperature was tracked over time at this fixed location throughout the experiment. In our numerical simulations we show that a good agreement with experiments can be obtained if the experimental configuration is replicated (i.e. fixing the interrogation region).This gives us confidence to report the overall kernel peak temperature using solid blue line as predicted by the CFD model. We find that a change of 500 K in terms of initial (post laser-pulse) plasma temperature profile in the model changes the fit of the experimental Rayleigh scattering radial profiles (shown in Fig. 8) by less than 5%. In terms of using the CFD output to correct for the change in hot-spot location (solid blue curve of Fig. 4), we find that the predicted temperature profiles (at plotted points of t ≥ 10 us) change by < 2% due to 500 K change of the initial (post laser-pulse) plasma temperature.

### Ignition testing

Combustion studies were performed in a chamber with central volume of ~ 0.195 L and two side arms of length ~ 20 cm with 2.54 cm diameter circular windows for optical access. Pressure traces were recorded using a piezoelectric transducer (PCB: 113B24) mounted flush on the inner wall of the chamber. At least ten pressure traces were collected for each equivalence ratio, and the results were averaged. The traces shown in Fig. 5-left are the average traces among those that ignited individually (with uncertainty band also due to igniting traces). The average combustion pressure traces due to all events at the given condition (whether or not ignited) were used to determine the lean limit and the combustion efficiency in each test case (Fig. 5-right). The rate of pressure rise is proportional to the rate of heat release. Thus, integrating the pressure curve over time gives the total heat release during the combustion event. The actual combustion efficiency is determined as the ratio of the apparent heat release and the total available chemical energy of the fuel: $\eta = Q/\left( {m_{fuel} \times LHV} \right)$, where Q is the total heat release obtained by integration, $m_{fuel}$ is the mass of fuel added in the reaction chamber and LHV is the lower heating value of the fuel. This calculation also takes into account the heat losses to the wall as well as the changes in the mixture’s specific heat ratio throughout the combustion process. A detailed description of the procedure can be found in one of our earlier works^47^.

Additionally, chemiluminescence images of the OH* radical were acquired with the same ICCD camera used in the scattering experiments. The electronically excited hydroxyl radical is generated during the combustion of hydrocarbon fuels through the chain branching reaction: CH + O_2_ = OH* + CO. The excited OH* emits light at ~ 310 nm as it relaxes to the ground state^48^. For CL imaging of this transition a 310 nm bandpass filter (Andover: 310FS10-50, FWHM: 10 nm) was placed in front of the ICCD.

The combustion experiments presented in this study were due to propane-air mixtures at initial pressure of 1 bar. Equivalence ratios ranging from ϕ~0.6–1.0 were tested with mixtures prepared inside the chamber based on partial pressures recorded from a gage (Omega DGP409) mounted downstream of the chamber valve. The equivalence ratio is defined as the ratio of the actual fuel/air ratio to the stoichiometric fuel/air ratio (so that ϕ = 1 corresponds to a stoichiometric mixture and leaner mixtures have ϕ < 1). After filling the chamber, but prior to measurements, we waited at least 10 min to allow thorough mixing of the fuel and air components. The chamber was evacuated and pumped down after each experiment to eliminate any effects of residual gases on subsequent experiments.
